# Selenium Organic Content Prediction in Jengkol (*Archidendron pauciflorum)* and Its Molecular Interaction with Cardioprotection Receptors PPAR-γ, NF-κB, and PI3K

**DOI:** 10.3390/molecules28103984

**Published:** 2023-05-09

**Authors:** Ayu Shalihat, Ronny Lesmana, Aliya Nur Hasanah, Mutakin Mutakin

**Affiliations:** 1 Department of Pharmaceutical Analysis and Medicinal Chemistry, Faculty of Pharmacy, Universitas Padjadjaran, Jl. Bandung Sumedang Km 21, Jatinangor, Sumedang 45363, Indonesia; 2 Physiology Division, Department of Biomedical Science, Faculty of Medicine, Universitas Padjadjaran, Jl. Bandung Sumedang Km 21, Jatinangor, Sumedang 45363, Indonesia

**Keywords:** organic selenium, cardioprotection, molecular interaction

## Abstract

Selenium (Se) is a trace mineral found in plants with a distinct sulfuric odor that is cardioprotective and reported to have low toxicity. West Java, Indonesia, has a variety of plants with a distinct odor that are consumed raw, such as jengkol (*Archidendron pauciflorum)*. This study is conducted to determine the Se content of jengkol using the fluorometric method, where the jengkol extract is separated, and the Se content is detected using high-pressure liquid chromatography (HPLC), combined with fluorometry. Two fractions with the highest Se concentration (A and B) are found and characterized using liquid chromatography mass spectrometry to predict the organic Se content by comparing the results with those in the external literature. The Se content of fraction (A) is found to be selenomethionine (*m*/*z* 198), gamma glutamyl-methyl-selenocysteine-(GluMetSeCys; *m*/*z* 313), and the Se-sulfur (S) conjugate of cysteine-selenoglutathione (*m*/*z* 475). Furthermore, these compounds are docked on receptors involved in cardioprotection. The receptors are peroxisome proliferator-activated receptor-γ (PPAR-γ), nuclear factor kappa-B (NF-κB), and phosphoinositide 3-kinase (PI3K/AKT). The interaction of receptor and ligan that has the lowest binding energy of the docking simulation is measured with molecular dynamic simulation. MD is performed to observe bond stability and conformation based on root mean square deviation, root mean square fluctuation, radius gyration, and MM-PBSA parameters. The results of the MD simulation show that the stability of the complex organic Se compounds tested with the receptors is lower than that of the native ligand, while the binding energy is lower than that of the native ligand based on the MM-PSBA parameter. This indicates that the predicted organic Se in jengkol, i.e., gamma-GluMetSeCys to PPAR-γ, gamma-GluMetSeCys AKT/PI3K, and Se-S conjugate of cysteine-selenoglutathione to NF-κB, has the best interaction results and provides a cardioprotection effect, compared to the molecular interaction of the test ligands with the receptors.

## 1. Introduction

In recent years, cardiovascular disease (CVD) has been the leading cause of death in the world [1]. Various triggers of the disease include other diseases, such as hypertension and diabetes, and lifestyle factors, such as a lack of exercise, smoking, and a high-fat diet [2]. The risk factors damage and cause the death of cardiomyocytes, which leads to CVD [3]. The cell survival processes associated with apoptosis or necrosis, inflammation, and autophagy [4] involve the peroxisome proliferator-activated receptor-γ (PPAR-γ; apoptosis and inflammation) [5], nuclear factor kappa-B (NF-κB; inflammation) [6], and phosphoinositide 3-kinase (PI3K/AKT; autophagy and apoptosis) [7], all of which play a role in heart cell survival.

Furthermore, due to their role in cell metabolism, several minerals, such as iron (Fe), zinc (Zn), copper (Cu), and selenium (Se), play major roles in a cardioprotective diet [8]. An Se-deficient diet can cause heart cell defects, leading to *Keshan* disease [9,10]. A previous study claimed that Se has a cardioprotective effect, preventing necrosis of heart cells [11], reducing apoptosis and autophagy [12], and reducing inflammation [13]. Investigation of the cardioprotective mechanism of Se as well as the signaling pathways involved is ongoing in the development of new therapeutic strategies [14]. The effects of Se in reducing apoptosis and autophagy via the PPAR-γ pathway [15], cardio- and endothelial cell protection via the PI3K/AKT pathway [16,17], and inflammation through activation of the NF-ΚB, which is a proinflammatory cytokine, have all been previously studied [18].

Some evidence shows that Se supplementation is beneficial for preventing chronic diseases and it is followed with the development studies of selenium as a dietary supplement from food sources [19] and plants [20]. The organic form of Se is often given as a supplement because it is less toxic [21], has better absorption, and has high bioavailability [22]. Organic Se is also known to have anti-inflammatory and cardioprotective effects [23], such as that of selenomethionine [24,25]. Various plants are natural sources of organic Se [26,27] wherein Se is transported via a sulfur (S) transporter and metabolized in the S pathway [28]. Previous studies have shown that the Se and S in plants compete for biochemical processes such as the assimilation of amino acids into essential proteins [29]. The S content of *Allium* sp. produces a characteristic odor and is found to contain selenocysteine and glutamyl-methyl-selenocysteine (GluMetSeCys) [30]. Jengkol (*Archidendron pauciflorum*) fruit seeds have a strong amino acid cysteine scent, which contains S [31]. The S contents of *Allium sativum* (garlic) [32] and jengkol, are 2.3% and 25.9%, respectively [33], which means that the Se content in jengkol is higher than that in garlic. Jengkol is often consumed raw and is part of the culture of West Java, Indonesia [34]. Therefore, it is interesting to know the characteristics of organic Se in jengkol as a candidate of organic Se dietary supplement’s food source and its correlation with cardioprotection through molecular interaction.

Currently, studies on the types of organic Se in jengkol and their molecular interaction with a cardioprotection receptor have not been carried out. In this study, we analyze Se and predict the characteristics of organic Se in jengkol using fluorometric analysis, followed by liquid chromatography mass spectrometry (LC-MS). The spectrum data obtained are matched with those from the external literature or previous studies. Meanwhile, to determine activity in heart protection, an in silico study is carried out on several cardioprotective receptors. The studies include molecular docking and molecular dynamic simulation through PPAR-γ, NF-ΚB, and AKT/PI3K.

## 2. Results

### 2.1. Jengkol Se Content Analysis

First, to determine the selenium concentration, a calibration standard was made with various concentrations of sodium selenite (Na_2_SeO_3_) which were calculated as selenium. The calibration standard series concentrations were 0, 0.05, 0.10, 0.20, 0.50, and 1.00 μmol/L (equation: y = 26,704x + 26,148; coefficient correlation: 0.999). The limits of detection and quantification obtained were 0.21 and 0.63 μmol/L, respectively. Jengkol was obtained from 15 cities or regencies in West Java, Indonesia, and their Se levels range from 27.3 to 498 ng/g. The highest level of selenium detected was in Kabupaten Subang. The results of the Se content of jengkol from West Java are shown in Table 1.

### 2.2. Separation of the Jengkol Extract Using HPLC Combined with Fluorometry

**Figure 1:**
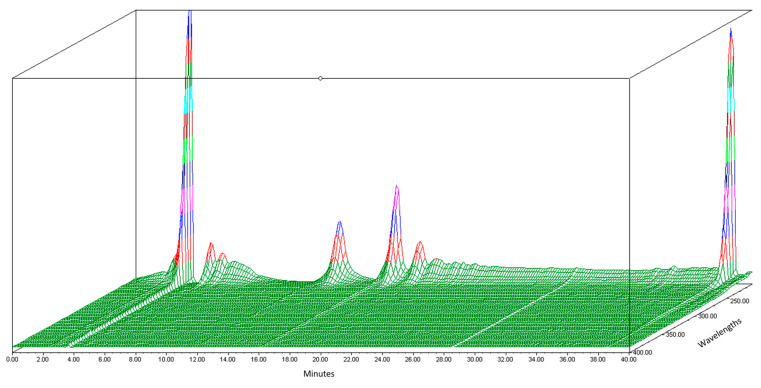
3D Chromatogram of jengkol extract using HPLC-PDA.

Before the organic Se characterization of jengkol, separation of the jengkol extract using HPLC based on elution time was performed. In this study, the HPLC-PDA Waters^®^ (photodiode array detector) was used to obtain a fingerprint 3D chromatogram for jengkol extract. The mobile phase used was a gradient elution system with water and ACN for 40 min. The chromatogram showed several peaks (Figure 1) with different maximum wavelengths. The separation solution was stored in the tube at elution times of 0–10 (0), 10–20 (A), 20–30 (B), and 30–40 (C) min. Separation was carried out to obtain the fraction with the most Se content, which was then characterized for its organic Se content using the fluorometric method.

**Figure 2:**
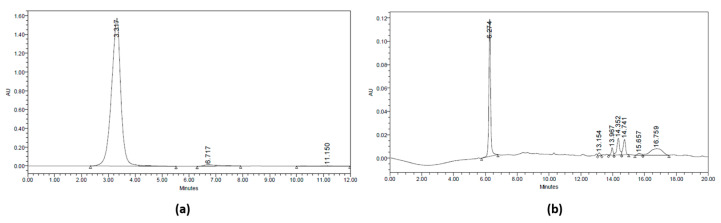
HPLC Chromatogram at 210 nm. (**a**) Fraction (A) of jengkol extract (10–20) and (**b**) fraction (B) of jengkol extract (20–30).

These fractions (Table 2) were identified, and their levels were calculated using the fluorometric method with 2,3-diaminonaphthalene reagent. The determination of selenium concentration in the separated fractions was carried out by calibration standard. The fluorometric method used the Se selenite as the standard with various concentrations of 0.1–1 μmol/L. The correlation coefficient (r) obtained was 0.96 and the equation y = 32,297x + 17,918. The fractions (A) and (B) had Se levels of 0.45 and 0.7 μmol/L, respectively, with fraction B having the highest Se concentration (Table 2). The 3D chromatogram was scanned at 210–400 nm. It shows four main peaks at 8.77, 13.11, 16.96, and 18.441 min of retention time, which had maximum wavelength at 375, 265, 273, and 267 nm, respectively (Figure 2).

Because the peaks in fraction (B) were more numerous, the HPLC results were separated again into a tube. Fraction (B) was collected at 0–10 (B1) and 10–20 (B2) min. A second separation was performed to reduce the amount of the compound in the solution. The collected fractions were put into a tube and freeze-dried to reduce the solvent for further analysis using LC-MS.

### 2.3. Characterization of Organic Se Using LC-MS

**Figure 3:**
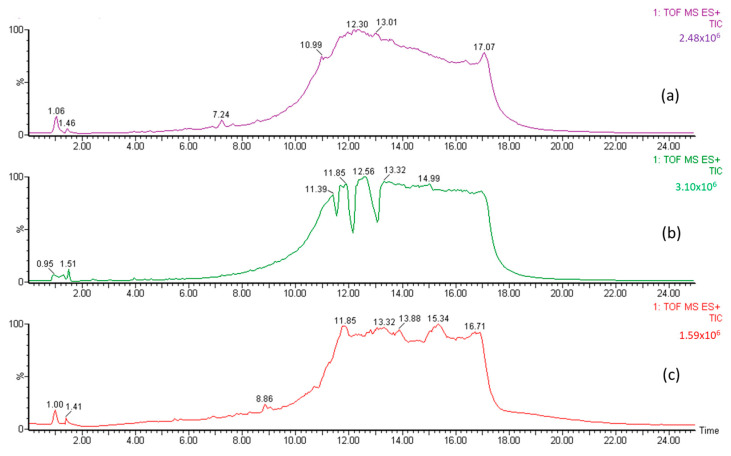
LC chromatogram for (**a**) fraction (A) of the jengkol extract, (**b**) fraction (B1) of the jengkol extract, and (**c**) fraction (B2) of the jengkol extract.

The A, B1, and B2 fractions were then analyzed using LC-MS to identify organic Se compounds based on their molecular weight. The chromatogram of jengkol fraction (Figure 3) shows a different peak and fingerprint. Each peak was analyzed using MS. The data collection on organic Se compounds was carried out on the basis of previously published scientific articles [27,35,36,37]. In addition, compound prediction was carried out using MS based on the possible elemental composition of the compound.

**Figure 4:**
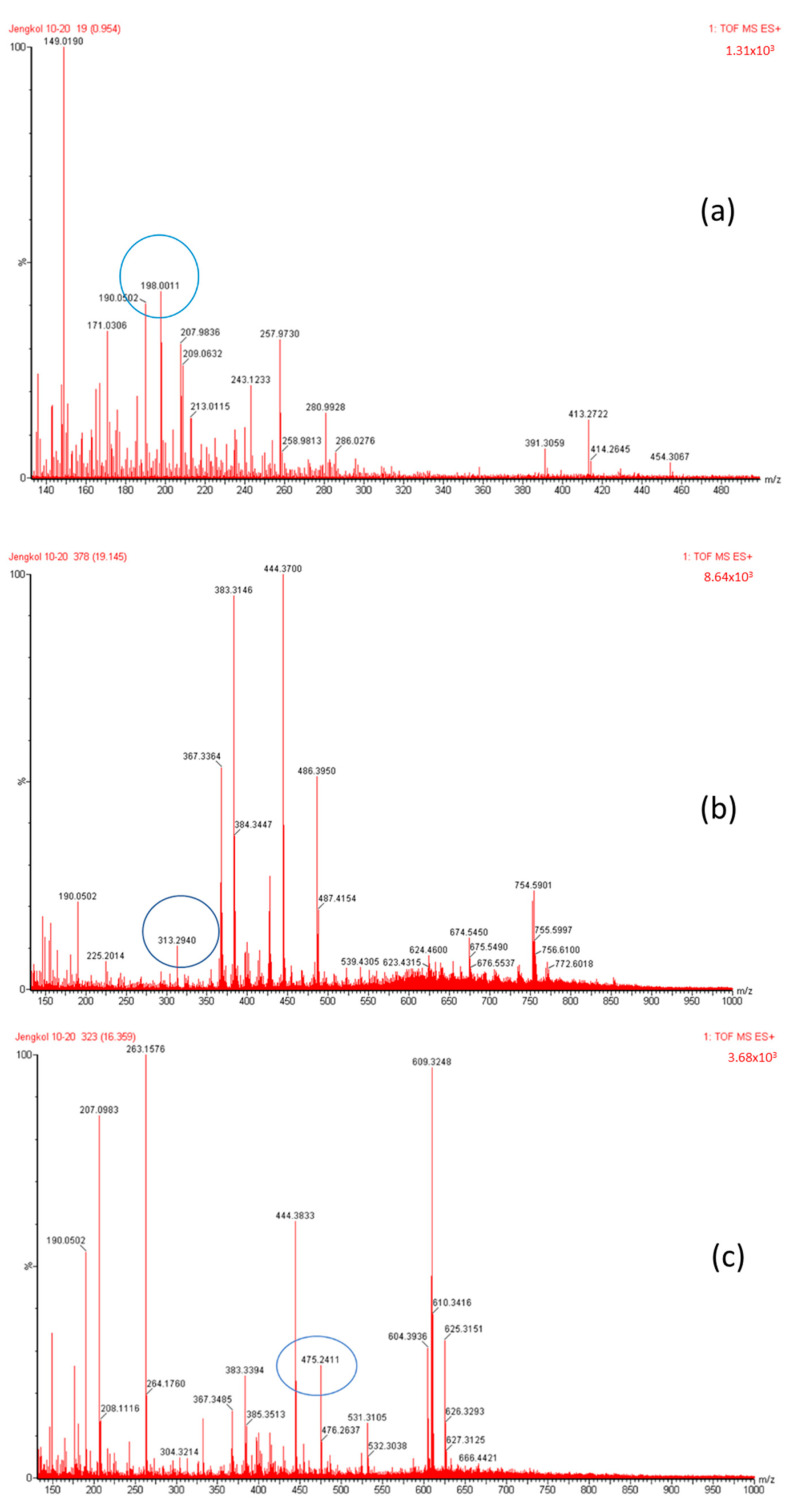
Mass spectrum fraction of the jengkol extract (A), i.e., (**a**) selenomethionine (*m*/*z* 198), (**b**) gamma-glu-MetSeCys (*m*/*z* 313), and (**c**) the Se-S conjugate of cysteine-selenoglutathione (*m*/*z* 475).

The organic Se compounds found in fraction (A) are selenomethionine [38], gamma-glu-MetSeCys [27], and the Se-S conjugate of cysteine-selenoglutathione [27], whose molecular weights, according to the literature, are *m*/*z* 198, *m*/*z* 313, and *m*/*z* 475, respectively, as shown in Figure 4. In the (B1) fraction are C_3_H_9_N_4_Se (*m*/*z* 181) and C_5_H_11_N_4_OSe (*m*/*z* 223), which are fragments of Se organic compounds. In the (B2) fraction, most of the compounds cannot be determined with the previous literature, so they were excluded from this study. The result of prediction compounds in fraction (A) was continued for molecular docking studies. The organic Se compounds based on the possible elemental composition of the compounds are shown in Table 3.

### 2.4. Docking Simulation of Organic Se in Jengkol to Cardioprotection Receptor

The molecular docking was performed with three ligand tests, which were the result of the LC-MS prediction of fraction (A) of the jengkol extract, i.e., between selenomethionine (Se01), gamma-glu-MetSeCys (Se02), and the Se-S conjugate of cysteine-selenoglutathione (Se03) and the cardioprotective receptors, PPAR-γ, NF-κB, and AKT/PI3K.

**Figure 5:**
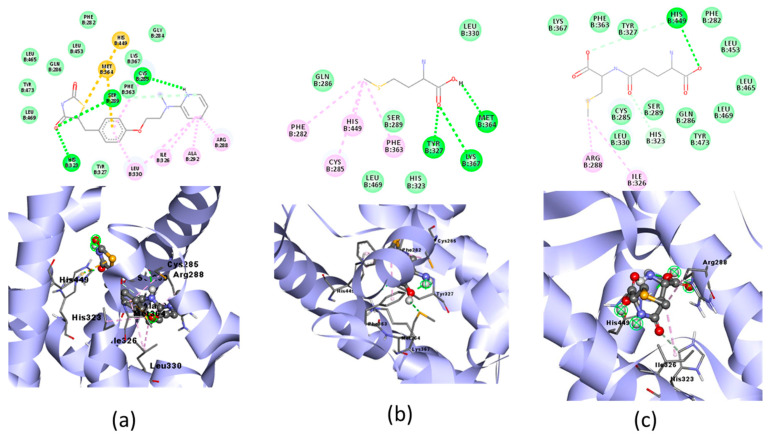
Molecular docking visualization of PPAR-γ and organic Se (**a**) the Se-S conjugate of cysteine-selenoglutathione (Se03), (**b**) selenomethionine (Se01) and (**c**) gamma-GluMetSeCys (Se02).

The result of the docking of the three organic Se compounds with PPAR-γ and their visualization are shown in Figure 5. The docking simulations in the three ligand tests were analyzed for binding energy, hydrogen bond, hydrogen bond distance, nearest amino acid residues, and other interactions (Table 4).

**Figure 6:**
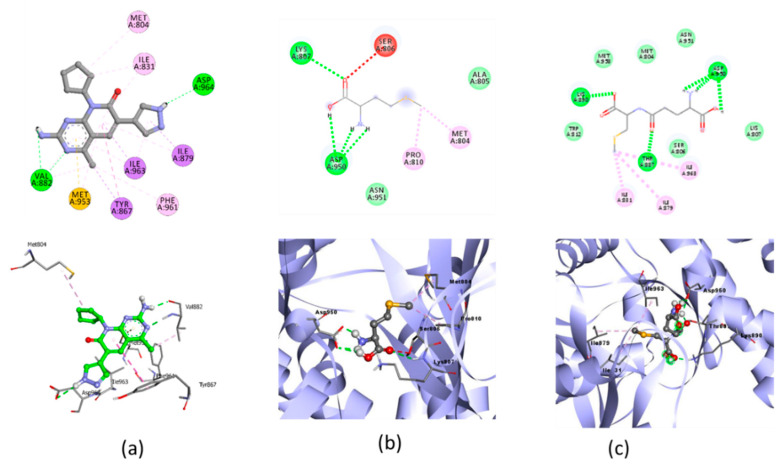
Molecular docking visualization of AKT/PI3K and (**a**) Native Ligand, (**b**) selenomethionine (Se01) and (**c**) Gamma-GluMetSeCys.

The molecular docking simulations of the native ligand with AKT/PI3K receptors are shown in Figure 6, and the result of the docking of the three organic Se compounds are written in Table 5.

**Figure 7:**
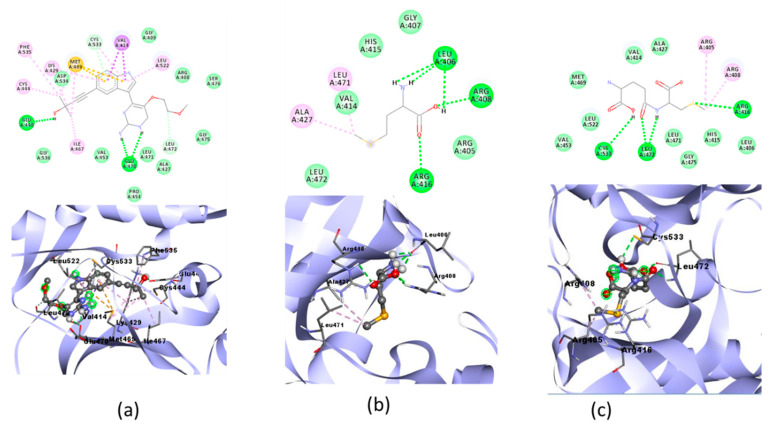
Molecular docking visualization of NF-ΚB and (**a**) Native Ligand, (**b**) selenomethionine (Se01) and **c**) gamma-GluMetSeCys (Se02).

The molecular docking simulations of NF-KB are shown in Table 6. The molecular docking visualization of the NF-KB receptor is shown in Figure 7.

### 2.5. Molecular Dynamic Simulation

#### 2.5.1. Root Mean Square Deviation (RMSD) and Root Mean Square Fluctuation (RMSF) Analysis of the Ligand–Receptor Complex

**Figure 8:**
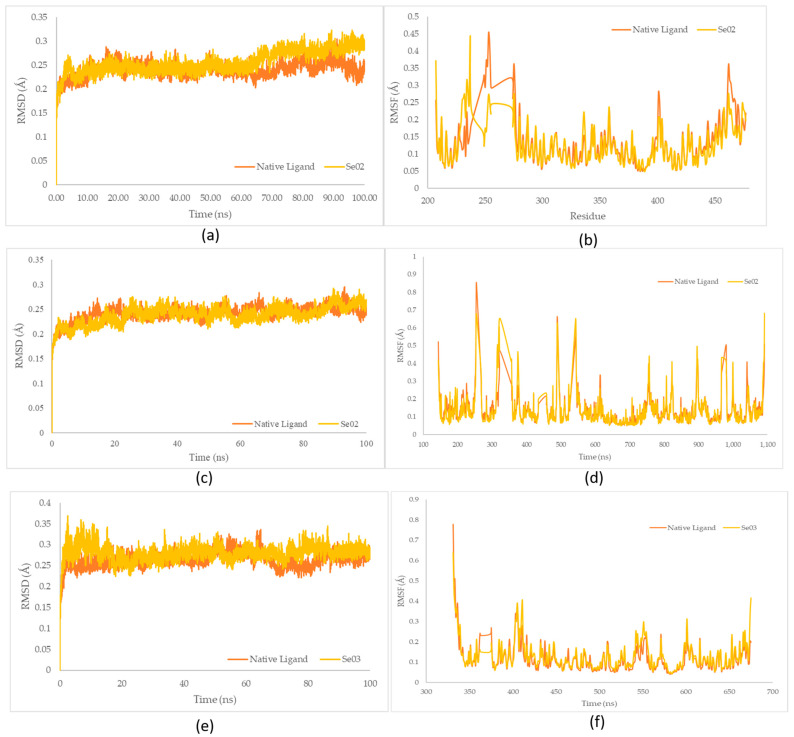
(**a**) RMSD complex of PPAR-γ; (**b**) RMSF value of native ligand–PPAR-γ (orange) and gamma-GluMetSeCys (Se02)–PPAR-γ (yellow) complexes; (**c**) RMSD complex of PI3K; (**d**) RMSF value of native ligand–PI3K (orange) and gamma-GluMetSeCys (Se02)–PI3K (yellow) complexes; (**e**) RMSD complex of NF-ΚB; (**f**) RMSF value of native ligand–NF-ΚB (orange) and Se-S conjugate of cysteine-selenoglutathione (Se03)– NF-ΚB (yellow) complexes.

The ligand–receptor complexes were analyzed using molecular dynamic simulations over a 100 ns simulation using GROMACS 2016. System stability over a 100-ns simulation was successfully measured via RMSD and RMSF (Figure 8).

The native ligand–PPAR-γ complex showed lower fluctuations than gamma-GluMetSeCys (Se02), with RMSD fluctuations of 0.240 Å and 0.255 Å, respectively. Furthermore, the gamma-GluMetSeCys (Se02)–PI3K complex showed low fluctuations compared with the native ligand, with an average increase in RMSD for each system at 0.237 Å and 0.240 Å, respectively. Meanwhile, the native ligand–NF-KB complex showed a similar decrease to the Se-S conjugate of cysteine-selenoglutathione (Se03), with an average RMSD fluctuation of 0.264 Å and 0.280 Å, respectively. The RMSD average values showed the lowest fluctuations for native ligands of the PPAR-γ and NF-KB receptors, while gamma-GluMetSeCys (Se02) had the lowest fluctuations for PI3K receptors.

#### 2.5.2. Solvent-Accessible Surface Area (SASA) Analysis

**Figure 9:**
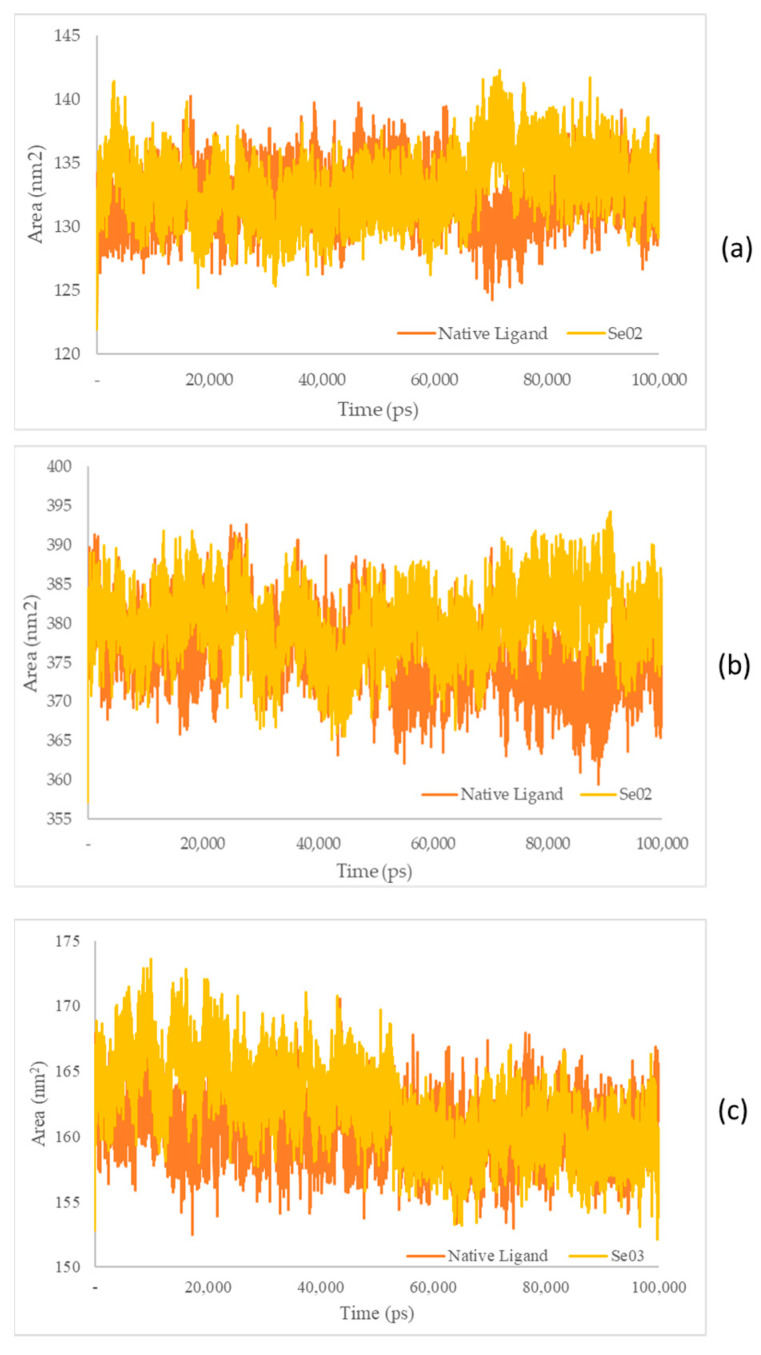
(**a**) SASA plot of the native ligand–PPAR-γ (orange) and gamma-GluMetSeCys (Se02)–PPAR-γ (yellow) complexes; (**b**) SASA plot of the native ligand–PI3K (orange) and gamma-GluMetSeCys (Se02)–PI3K (yellow) complexes; (**c**) SASA plot of the native ligand–NFκB (orange) and the Se-S conjugate of cysteine-selenoglutathione (Se03)–NF-KB (yellow) complexes.

The identification of SASA was carried out to predict conformational changes of proteins during the simulation that were accessible to water molecules. SASA value estimates the increased of computational complexity and accuracy, as well as the knowledge-based environmental of free energy potential based on the SASA values [39]. SASA was used to analyze over 100 ns of simulations of the MD trajectory, which is shown in Figure 9.

#### 2.5.3. Radius Gyration (Rg) Analysis

**Figure 10:**
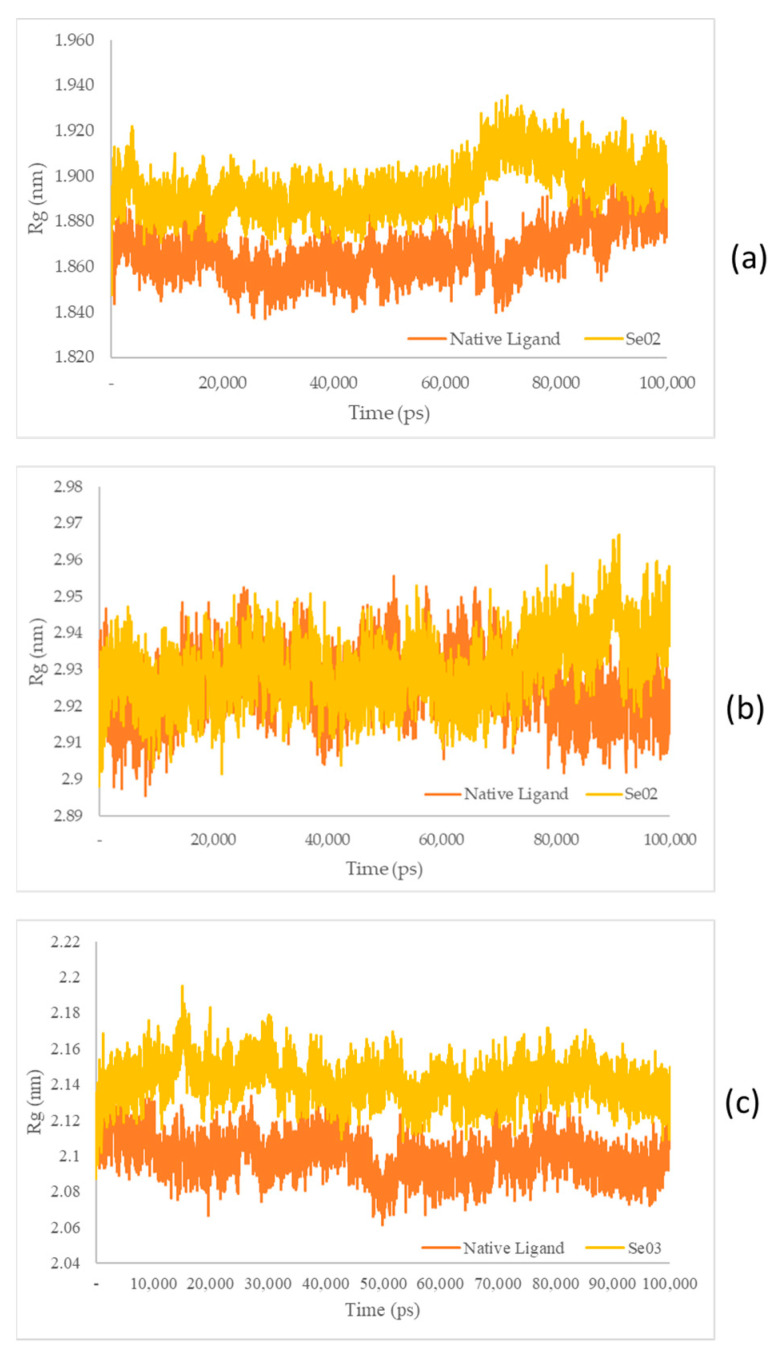
(**a**) Rg plot of the native ligand–PPAR-γ (orange) and gamma-GluMetSeCys (Se02)–PPAR-γ (yellow) complexes; (**b**) Rg plot of the native ligand–PI3K (orange) and gamma-GluMetSeCys (Se02)–PI3K (yellow) complexes; (**c**) Rg plot of the native ligand–NF-ΚB (orange) and the Se-S conjugate of cysteine-selenoglutathione (Se03)–PI3K (yellow) complexes.

The measurement of the Rg aims to identify the stability of the complex in its folded or unfolded form during the simulation [40]. The Rg plots of the molecular dynamic simulation of the three receptors and each test ligand are shown in Figure 10.

#### 2.5.4. MM-PBSA Binding Free Energy Calculations

The binding free energy of the molecular dynamic trajectories of the system complexes was calculated using the MM-PBSA method from the 0–100 ns timestep (Table 7).

## 3. Discussion

### 3.1. Analysis of the Se Content in Jengkol

A fluorometric method was performed for the formation of the complexes of piazselenol, which is from selenite and 2,3-diaminonaphthalene. This method was recommended in the AOAC 1996 compendium for Se determination [41]. The advantages of the fluorometric method are that it can be used to measure low sample concentrations and is more selective and sensitive. The fluorometric method makes it possible to reach detection limits of up to ppm–ppb units [42,43].

The highest level of Se from jengkol, detected in Kabupaten Subang, West Java, was 498 ng/g, which is higher than that of garlic (69.2 ng/g) from previous studies of the same province [44]. In another study from Turkey, the Se levels in onions and garlic were 24 ng/g and 15 ng/g, respectively [45], showing that jengkol has a greater Se content and is a potential source of Se supplementation. The procedure was continued with the characterization of organic Se based on the fraction with the highest Se content.

### 3.2. Separation of the Jengkol Extract and Characterization of Organic Se

Fractions (A) and (B) have compounds with lower molecular weights, and their retention times show higher polarity because the more carbon and hydrogen atoms they have, the less polar they are [46]. In a previous study, the selenomethionine (Se01), gamma-GluMetSeCys (Se02) and Se-S conjugate of cysteine-selenoglutathione (Se03) were seleno-compounds that are found in sunflower, radish, and onion sprouts by MS [27]. Selenomethionine and gamma-GluMetSeCys are also found in garlic (*A. sativum*) [37]. An in vitro study of H9C2 cardiac myoblasts found the potential benefit of selenomethionine with limited efficacy as an agent for treatment of heart attacks [24]. Contrary to claims that Se has a protective effect, cardiotoxic studies in zebrafish have shown that selenomethionine has a negative effect on the hearts of fish [47]. But until now, there has been no study of the interaction between selenomethionine, gamma-GluMetSeCys, and the Se-S conjugate of cysteine-selenoglutathione and receptors that affect cardioprotection.

### 3.3. Docking Simulation of Organic Se in Jengkol to a Cardioprotective Receptor

#### 3.3.1. Preparation of Protein Receptor and Validation

PPAR-γ, which belongs to the superfamily of nuclear receptors and serves as a ligand-inducible transcription factor, has been studied in CVDs [48]. Recent studies have explained that PPAR-γ activation can prevent an inflammatory response in cardiac tissue [49]. To determine whether the docking molecular simulation system was valid, validation was carried out by redocking native ligands. The validation obtained with the redocking of the native ligand (rosiglitazone) to PPARγ receptors (PDB:2PRG) was 1.91 Å, which met the requirement (<2 Å). The Gibbs free energy (ΔG) obtained −9.37 kkal/mol, and the hydrogen bond was at CYS^285^, SER^289^, HIS^323^. The acidic head group is crucial for PPAR activation. It forms an H-bonding network with a part of the PPAR that mainly contains the critical polar residues. The carbonyl groups of rosiglitazone create hydrogen bonds with HIS^449^ and HIS^323^ [50].

The molecular docking validation of the native ligand to AKT/PI3K receptors obtained 0.635 Å, which met the requirement (<2 Å). The Gibbs Free energy (ΔG) obtained −8.7 kkal/mol, and the hydrogen bond was at VAL^882^ and ASP^964^. NF-κB validation was 1.732 Å and met the requirements (<2 Å) [51]. The Gibbs free energy (ΔG) obtained −9.71 kkal/mol, and the hydrogen bond was at GLU^440^ and GLU^470^.

#### 3.3.2. Docking Simulation

PPAR-γ is a key regulator in maintaining energy homeostasis [52] and functions as a ligand-induced transcription factor whose activation inhibits cardiac tissue inflammatory responses and minimizes ischemic pathological damage to the heart [53]. The test ligand was docked with PPAR-γ; Se02 had the lowest free binding energy (−4.44) compared with Se01 and Se03, which were−3.99 and −3.32, respectively, but was higher when compared with the native ligand, which was −9.37. SE02 created hydrogen bonds with HIS^449^ and had a greater number of the same interaction sites as that of the native ligand, such as LEU^330^, LEU^453^, LEU^465^, LEU^469^, PHE^282^, PHE^363^, TYR^327^, TYR^473^, HIS^323^, ARG^288^, and ILE^326^. For the hydrogen bond and hydrogen bond distance, the three test compounds did not have similarities with the native ligands. Therefore, the SE02 compound was chosen for its molecular dynamics with the PPAR-γ receptor.

Oxidative stress is characterized by a decreased activation of the PI3K/AKT pathway in the apoptosis of various cells [54]. Research on Se-deficient heart cells showed inhibition of the PI3K/AKT pathway [17]. The test ligand was docked with AKT/PI3K, and the result showed that SE02 had the lowest free binding energy compared with SE01 and SE03, which were −5.04, −4.5, and −4.67, respectively. Compared with the native ligand, the three test ligands had a higher bond-free energy at −8.7. SE02 had a greater number of the same interactions as the native ligand, such as MET^804^, ILE^831^, ILE^879^, and ILE^963^. For the hydrogen bond and hydrogen bond distance, the three test compounds did not have similarities with the native ligands. Therefore, the SE02 compound was chosen for its molecular dynamics with the AKT/PI3K receptor.

The protective effect of Se on inflammation associated with oxidative stress can also be seen from the activation of NF-ΚB, which is a proinflammatory cytokine [18]. In the molecular docking of the three organic Se compounds, SE03 had the lowest free binding energy compared with SE01 and SE02, which were −5.56, −4.86, and −5.27, respectively. Compared with the native ligand, the three test ligands had a greater bond-free energy. SE03 also had a greater number of the same interactions as the native ligand, such as GLY^475^, LEU^472^, ASP^534^, SER^476^, and GLU^470^. SE03 had the same hydrogen bond as native ligands on GLU^470^ with a larger hydrogen bond distance, and had the nearest amino acid residue, which was the same as that of the native ligand, specifically GLU^470^. SE01 and SE02 did not have the same hydrogen bonds or closest amino acid residues as the native ligand. Therefore, the SE03 compound was chosen for its molecular dynamics with the NF-κB receptor.

### 3.4. Molecular Dynamic Simulation

RMSD analysis was used to assess the stability of the complex over time, and RMSF analysis was used to assess the fluctuation and stability per amino acid [55]. The native ligand and the best test ligand result of docking stimulation were perform with molecular dynamics . After that the complex stability of Se02 also compared to the native ligand which inhibited PPAR-γ receptors. Amino acid fluctuations of the two receptor complex systems analyzed using RMSF showed similar patterns in all regions for the PPAR-γ receptor residue numbers: 207, 237, 253, 275, 401, and 462; and for the PI3K receptor: 144, 255, 323, 490, 544, 980, and 1092. The residue values of NF-ΚB receptors (331, 405, 411, 601, and 675) compared with the PPAR-γ, PI3K, and NF-KB receptors showed higher fluctuations than other residues. These residues were visible on the amino acid chain which was responsible for the loop region.

The SASA receptor ligand–PPAR-γ complex was revealed. In the graph, the native ligand shows similarly low fluctuations compared with Se02, with values of 132.33 nm^2^ and 133.21 nm^2^, respectively. The SASA for the PI3K–ligand–receptor complex shows a graph that has the same lower fluctuation value as Se02, with an average value of 375.84 and 380.09 nm^2^ for the native ligand and Se02, respectively. SASA for the NF-ΚB–ligand–receptor complex shows a graph that has similar decreased-fluctuation values compared to Se03, with an average value of 160.62 and 162.27 nm^2^ for the native ligand and Se03, respectively. Lower SASA values were given by the native ligand, followed by the test ligand. A lower SASA value indicates a more stable complex system [56]. This analysis correlates with the RMSD value, which showed that the native ligand had better stability with the receptor than the tested ligand.

The Rg plots show a characteristic similarity between the protein–native ligand complex as well as the protein–exposed test ligands (SE02 and engkolSE03). Analysis of the stability of the native ligand–PPAR-γ complex shows a graph with a low stability value against SE02, with an average Rg value of 1.86 and 1.89 nm for the native ligand-PPAR-γ complex and SE02, respectively. Rg in the native ligand–PI3K complex, which yielded better stability values than SE02, yielded Rg values of 2.92 nm and 2.93 nm for the native ligand–PI3K complex and SE02, respectively. Analysis of Rg on the native ligand–NF-ΚB yielded good stability values for engkolSE03, with the average values of engkol being 2.09 and 2.14 nm, respectively. The native ligand that interacted with the receptor protein showed a significant similarity and a stable folding structure compared with the protein–native ligand complex.

Van der Waals and electrostatic and SASA energies in both complex systems showed negative values, whereas the polar solvation energy showed a positive value. These results, in both system complexes, indicated the polar solvation energy terms opposed the binding, whereas van der Waals and the electrostatic and SASA energies favored the binding. The total free energy of the ligand bonds showed various values. SE02 provided the lowest binding free energy compared with the native ligand. SE02 showed a total binding energy of −129.919 KJ/mol, while that of the native ligand was −69.565 KJ/mol. MM-PBSA analysis showed that SE02 had the strongest affinity to PPAR-γ. Furthermore, Se02 9 (−82.767 KJ/mol) provided the lowest binding free energy compared with the native ligand (−20.011 KJ/mol), which indicated that SE02 had the strongest affinity for PI3K receptors. Meanwhile, SE03 (−99.091 KJ/mol) provided the lowest binding free energy compared with the native ligand (−69.565 KJ/mol), which indicated that SE03 had the strongest affinity for the NF-κB receptor.

## 4. Materials and Methods

### 4.1. Materials

Samples of jengkol were obtained from several markets in West Java, using the cluster sampling method. The stationary phase used for HPLC was column C18 (250 × 4.6 mm, 5 µm) and for that of LC-MS was column C18 1.7 µm (2.1 × 100 mm). Chemical materials include 2,3-diaminonaphthalene (Sigma-Aldrich^®^, St. Louis, MO, USA), sodium selenite (Na_2_SeO_3_; Sigma-Aldrich^®^), acetonitrile (ACN; Merck^®^ (St. Louis, MO, USA) HPLC grade)), methanol (Merck^®^ HPLC Grade), cyclohexane (Merck^®^ Analysis Grade), nitric acid (Merck^®^ Analysis Grade), perchloric acid (Merck^®^ Analysis Grade), hydrochloric acid (HCl; Merck^®^ Analysis Grade), and Na-EDTA (Merck^®^ Analysis Grade). The analysis of molecular docking was performed using Auto dock 1.2.6, BIOVIA Discovery Studio Visualizer^®^ (San Diego, CA, USA), ChemDraw 8.0. The molecular dynamic simulation was performed using GROMACS 2016.3.

### 4.2. Standard and Sample Preparation

A standard solution stock of 200 µM Na_2_SeO_3_ was dissolved in 0.1 N HCl. A standard curve was prepared via dilution with various concentrations of 0.05–1 µM.

Jengkol samples for Se content analysis were crushed and weighed (400 mg), digested with nitric acid and perchloric acid (2:1), and then placed into a glass tube. The sample was destructed at 50–190 °C for 8 h in a tube heating block. After cooling, 0.5 mL of 10 N HCl was added, then heated again for 20 min at 150 °C.

As for characterization, the sample was crushed and weighed (20 g) to be extracted with 40 mL chloroform and then centrifuged. The supernatant was taken, evaporated at 50 °C, and dissolved with 10 mL ACN.

### 4.3. Analysis of the Se Content in Jengkol

Se forms a complex with 2,3-diaminonaphthalene by adding 0.1 mL of 0.1 N EDTA into sample tubes, standards, and blank. An amount of 0.1% of Thymol blue in ethanol was added with 25% ammonium hydroxide in an ice water bath. After color change occurred, 2N HCl was added to obtain a reddish color. Then, 1 mL of 0.1 N HCl and 0.1 mL of 2,3-diaminonaphthalene solution was added at 50 °C for 10 min. The extract was mixed with cyclohexane, shaken vigorously for 2 min, and centrifuged for 5 min at 2500 rpm. The cyclohexane part was pipetted and placed on 96-well plates (250 µL per well). The fluorometric intensity of cyclohexane was measured at the excitation wavelength of 378 nm and emission at 525 nm [57].

### 4.4. Separation of the Jengkol Extract Using HPLC Combined with the Fluorometric Method

The stationary phase used was reverse-phase HPLC, namely column C18 (250 × 4.6 mm, 5 µm). The elution system used a gradient system with the polar mobile phase. In the early stages, elution was carried out with variations in the percentage (%) of ACN and water concentration 20–80% for 40 minutes (Table 8). The sample was dissolved at a concentration of 100 ppm, filtered (0.45 µm PTFE membrane filter), and placed into a vial. Then, 20 µL of the sample solution was injected into the HPLC system and measured at a wavelength of 210 nm. The peaks on the chromatogram were observed for retention time and peak height. To identify Se, the results were collected by its separation time every 10 min and then measured using the fluorometric method.

### 4.5. Characterization of Organic Se Using LC-MS

The fraction that had been separated using HPLC was then injected into the LC-MS, which operates using the reverse-phase method (column C18 1.7 µm (2.1 × 100 mm)). The elution system uses a gradient system with the mobile phase water with 0.01% formic acid aIACN 5%–95% in 25 min, 0.2 mL/min flow rate, and 5 µL volume injection.

MS was performed using ESI-Quadrupole-Tof (Q-Tof MS Xevo, Waters (Milford, MA, USA) with a microchannel plates detector. The MS system was Capillary (kV) 3.0, Sampling Cone 20.0, Extraction Cone 2.0, Source Temperature 100 °C, Desolvation Temperature 250 °C, Cone Gas Flow 50.0 (L/Hr), Desolvation Gas Flow (L/Hr) 500.0, Purge Gas Flow (L/Hr) 500.0, Collision Energy 2.0 (eV), and Detector Voltage 2000 V. The chromatogram of each fraction along with the peaks and retention time were recorded, then it was continued with MS. The mass spectra were observed and compared with the literature [27,35,38].

### 4.6. Docking Simulation of Organic Se in Jengkol

#### 4.6.1. Preparation of the Ligand Structure

The two-dimensional structure of organic Se which was the predicted in jengkol obtained from PubChem. The ligands are shown in Table 9. The structure was then converted into a three-dimensional (3D) structure using ChemDraw 8.0.

#### 4.6.2. Preparation of the Protein Receptor

High-resolution crystal structures of the PPAR-γ ligand binding domain complexed with rosiglitazone [PDB ID: 2PRG] were retrieved as receptor proteins for molecular docking-based binding studies. The 3D structure of the protein was downloaded into the PDB file format. Co-crystallized ligands, native ligands, and solvent molecules were removed from the complex. Hydrogen atoms were added to protein atoms, and their positions were optimized by energy minimization. Preparation of the receptor native ligands was carried out using AutoDock Tools 1.5.6 to obtain the position of the grid box and determine the spatial shape and coordinates of the docking material [58]. The procedure was the same for the NF-κB receptor crystals (PDB ID: 4IDV) and AKT/PI3K (PDB ID: 4FA6).

#### 4.6.3. Validation of the Molecular Docking Method

Molecular docking validation was carried out using AutoDock Tools 1.5.6 by redocking a native ligand against a target protein that had had its natural ligand removed. Water ligands contained in the protein receptor were removed by adjusting the position of the grid box on the native ligand, which was then docked. The process was carried out to determine the RMSD value; native ligand redocking was considered successful if the RMSD value was <2 Å [51], which was obtained by looking at the overlay of the native ligand that was separated before docking, and the redocked validation of native ligand using Discovery Studio Visualizer.

#### 4.6.4. Docking Simulation

Optimization of the 3D structure of the native ligand and ligands was carried out using Chem3D Ultra 8.0 and the MM2 semi-empirical computational method. The calculation was performed by optimizing the geometry at the minimum energy of the 3D structure.

The docking method was carried out by tethering each ligand to each receptor using the pdbqt format and the coordinates of the grid box size for PPAR-γ: x, y, and z (59.415, −5.607, and 42.406); AKT/PI3K (4FA6): x, y, and z (44.503, 14.952, and 31.315); and NF-ΚB (4IDV): x, y, and z (16.109, 13.917, and 87.361), with 50 total docking poses. Each ligand was in a stable state, and interactions with biomacromolecules were in a rigid state. The docking results obtained in the form of binding energy values and chemical interactions, such as hydrogen bonds, hydrophobic interactions, and bond distances, were visualized using Discovery Studio Visualizer.

### 4.7. Molecular Dynamic Simulation

The molecular dynamic simulation was carried out using organic Se with the lowest binding energy results from the molecular docking for PPAR-γ, AKT/PI3K, and NF-κB. Molecular dynamic simulation was performed using the GROMACS 2016.3 software with the AMBER99SB-ILDN force field [59]. Topology and ligand parameters were made using ACPYPE [60]. The electrostatic force over a distance was determined by the particle mesh Ewald method [61]. Neutralization of the system was carried out by adding Na+ and Cl− ions. Solvation was carried out using the TIP3P water cube model. The simulation preparation stage included the minimization step, heating to 310 K, temperature equilibration, and pressure equilibration. Furthermore, 100 ns for the production of molecular dynamics was performed with a 2-fs timestep. After the simulation, generalized RMSD (g_rmsd), RMSF (g_rmsf), and Rg (g_rg) functions were calculated. Post-molecular dynamic simulation analysis was performed by calculating the SASA to detect the direction and amplitude of the dominant motions, MM-PBSA, RMSD, and RMSF.

## 5. Conclusions

The determination of Se content and the prediction of organic compounds in jengkol were successfully carried out. Se01, Se02, and Se03 docked to three cardioprotective receptors. The results of docking with the lowest binding free energy were followed by molecular dynamic simulations using parameters such as RMSD, RMSF, Rg, and MM-PSBA. The stability of the native ligand bond was better compared with the test ligand based on parameters such as RMSD, RMSF, and Rg. Meanwhile, the total binding energy of the test ligand was better than that of the native ligand based on MM-PSBA. Based on the molecular interactions, the predicted organic compound in jengkol, i.e., gamma-GluMetSeCys to PPAR-γ and AKT/PI3K, and the Se-S conjugate of cysteine-selenoglutathione to NF-κB, has the best interaction results and provides a cardioprotection effect, compared to the molecular interaction of test ligands with the receptors. However, further research needs to be carried out to support this insilico study such as apurification method of organic selenium that are specific and sensitive and also activity studies to cardio-protection. In addition, administration of selenium as a dietary supplement in CVD patients has been observed [62], although recent clinical studies show differences in the effect of selenium supplementation on CVD [63,64]. The development of selenium as a functional food from natural sources continues to be studied [65,66]. The results of this study can provide an overview of jengkol as a candidate for a natural source of organic selenium for cardioprotection supplementation.

## Figures and Tables

**Table 1:** Se content of Jengkol from West Java, Indonesia.

City or Regency	Se Content (ng/g)	SD
Kabupaten Bandung Barat	48	24.1
Kabupaten Kuningan	44.1	10.9
Kota Banjar	246.9	19
Kota Bogor	27.3	23.9
Kabupaten Purwakarta	264	31.1
Kabupaten Subang	498	66.6
Kabupaten Sukabumi	298.2	15.2
Kabupaten Garut	161.8	25.6
Kota Cimahi	35.0	6.5
Kota Bandung	92.1	9.7
Kota Tasikmalaya	74.9	51.2
Kabupaten Tasikmalaya	341.4	3.3
Kabupaten Bekasi	133.9	44.7
Kabupaten Sumedang	187.7	86.0

**Table 2:** Se concentration in jengkol extract fractions.

Code	Fraction	Intensity	Se Concentration (µmol/L)
0	0–10	19,280	0.09648
A	10–20	31,105	0.45070
B	20–30	40,453	0.70181
C	30–40	15,393	−0.02825

**Table 3:** Prediction of molecular formula based on (*m*/*z*) in the jengkol extract fraction.

Fraction	Molecular Weight (*m*/*z*)	Retention Time	Molecular Formula	Organic Se	Reference
**A**	198	0.95	C_5_ H_12_NO_2_Se	selenomethionine (C_5_H_12_O_2_Nse+)	[27,38]
	313	19.14	(−)	gamma-GluMetSeCys (C9H17O5N2Se+)	[27]
	609	16.36	C_26_H53N_6_O_5_Se	(−)	
	475	16.36	C_18_ H_43_N_4_O_5_Se	C_13_H_23_O_8_N_4_Sse+	[27]
**B1**	181	1.31	C_3_H_9_N_4_Se	C_5_H_9_O_2_Se+	[27]
	223	1.31	C_5_H_11_N_4_Ose	C_6_H_10_NO_3_Se+	[35]
	267	2.3	C_12_H_15_N_2_Se	(−)	
**B2**	384	1.05	C_13_H_5_N_4_O_9_Se	(−)	
	265	1.05	C_2_H_13_N_6_O_4_Se	(−)	
	779	6.88	C_3_4H_63_N_14_O_2_Se	(−)	
	761	7.24	C_38_H_64_N_7_O_4_Se	(−)	

(−) has not been determined.

**Table 4:** Molecular docking of PPAR-γ and selenomethionine, gamma-GluMetSeCys, and the Se-S conjugate of cysteine-selenoglutathione.

Code	Compound	Binding Energy (kkal/mol)	Hydrogen Bond Distance (Ǻ)	H-Bond Interactions	Nearest Amino Acid Residue(s)	Other Interactions
N	Native ligand	−9.37	2.17, 1.94, and 2.20	CYS^285^, SER^289^, and HIS^323^	SER^289^, CYS^285^, HIS^323^, ARG^288^	PHE^282^, PHE^363^, GLY^284^, TYR^327^, TYR^473^, LYS^367^, LEU^330^, LEU^453^, LEU^465^, LEU^469^, GLN^286^, HIS^323^, HIS^449^, ILE^326^, ALA^292^, ARG^288^, MET^364^
Se01	Selenomethionine	−3.99	2.11, 2.48, and 2.83	TYR^327^, MET^364^, and LYS^367^	TYR^327^, MET^364^, LYS^367^, PHE^282^	LEU^330^, LEU ^469^, GLN^286^, PHE^282^, PHE^363^, SER^289^, HIS^323^, HIS^449^, CYS^285^
Se02	Gamma-GluMetSeCys	−4.4	3.32	HIS^449^	HIS^323^, HIS^449^	LEU^330^, LEU^453^, LEU^465^, LEU^469^, PHE^282^, PHE363, TYR^327^, TYR^473^, HIS^323^, CYS^285^, SER^289^, GLN^286^, ARG^288^, ILE^326^, LYS^367^
Se03	Se-S conjugate of the cysteine-selenoglutathione	−3.32	1.96, 1.96, 2.12, and 2.70	GLY^284^, SER^342^, ILE^326^, SER^289^	GLY^284^, SER^342^, ILE^326^, SER^289^, CYS^285^	ILE^325^, ILE^341^, LEU^330^, LEU^333^, LEU^340^, MET^329^, MET^334^, MET^364^, PHE^363^, LYS^367^, TYR^327^, GLN^286^, VAL^339^, CYS^285^, ALA^292^, ARG^288^

**Table 5:** Molecular docking of AKT/PI3K and selenomethionine, gamma-GluMetSeCys, and the Se-S conjugate of cysteine-selenoglutathione.

Code	Compound	Binding Energy (kkal/mol)	Hydrogen Bond Distance (Ǻ)	H-Bond Interactions	Nearest Amino Acid Residue (s)	Other Interactions
N	Native ligand	−8.7	1.82	VAL^882^	VAL^882,^ TYR^867^	MET^804^, ILE^831^, TYR^867^, ILE^879^, ILE^963^MET953
Se01	Selenomethionine	−4.5	2.65 and 1.59	LYS^807^ and ASP^950^	SER^806^, LYS^807^, and ASP^950^	MET^804^, ALA^805^, SER^806^, PRO^810^, ASN^951^
Se02	Gamma-GluMetSeCys	−5.04	2.06, 1.70, 1.77	THR^887^, LYS^890^, and ASP^950^	THR^887^, LYS^890^, ASP^950,^ and ILE^831^	MET^804^, SER^806^, LYS^807^, TRP^812^, ILE^831^, ILE^879^, ASN^951^, MET^958^, ILE^963^
Se03	Se-S conjugate of cysteine-selenoglutathione	−4.67	2.47, 1.93, and 1.83	MET^804^, LYS^890^, and ASP^950^	ASP^950^, LYS^890^, MET^804^, LYS^833^	SER^806^, PRO^810^, TRP^812^, ILE^831^, LYS^833^, ILE^879^, THR^887^, ILE^963^

**Table 6:** Molecular docking of NF-ΚB and selenomethionine, gamma-GluMetSeCys, and the Se-S conjugate of cysteine-selenoglutathione.

Code	Compound	Binding Energy (kkal/mol)	Hydrogen Bond Distance (Ǻ)	Interactions H-Bond	Nearest Amino Acid Residue(s)	Other Interactions
N	Native ligand	−9.71	1.86 and 1.82	GLU^440^ and GLU^470^	GLU^440^, GLU^470^, ILE^467^	PHE^535^, LYS^429^, CYS^444^, ILE^467^, LEU^522^, VAL^414^, CYS^533^, LEU^472^, PRO^454^, VAL^453^, ALA^427^, LEU^471^, GLY^536^, GLY^475^, GLY^409^, ASP^534^, ARG^408^, SER^476^
Se01	Selenomethionine	−4.86	2.00, 1.78, and 2.19	LEU^406^, ARG^408^, and ARG^416^	ALA^427^, ARG^408^, LEU^406^, ARG^416^	GLY ^407^, HIS^415^, ARG^405^, VAL^414^, LEU^472^, ALA^427^, LEU^471^
Se02	Gamma-GluMetSeCys	−5.27	3.07, 1.85, and 2.85	ARG^416^, LEU^472^, and CYS^533^	LEU^472^, CYS^533^, ARG^416^, ARG^408^	ALA^427^, VAL^414^, LEU^471^, HIS^415^, LEU^406^, GLY^475^, MET^469^, LEU^522^, VAL^453^, ARG^405^, ARG^408^
Se03	Se-S conjugate of cysteine-selenoglutathione	−5.56	1.99, 3.32, 1.99, 2.62, 2.10, and 2.36	GLU^470^, CYS^533^, ASN^520^, ASP^519^, GLN^479^, and ARG^408^	ASN^520^, GLU^470^, GLN^479^	VAL ^453^, MET ^469^, ALA^427^, GLY^475^, LEU^522^, LEU^472^, ARG^416^, GLU^413^, ASP^534^, SER^410^, LEU^471^, SER^476^, GLY^409^, VAL^414^

**Table 7:** MM-PBSA energy summary of ligand–receptor during the 100 ns simulation.

Receptor	Ligand	Van Der Waals Energy (KJ/mol)	Electrostatic Energy (KJ/mol)	Polar Solvation Energy (KJ/mol)	SASA Energy (KJ/mol)	Total Binding Energy (KJ/mol)
PPAR-γ	Native ligand	−139.618 ± 11.007	−34.531 ± 25.211	120.493 ± 26.547	−15.909 ± 0.639	−69.565 ± 13.830
	Se02	−255.404 ± 11.487	−50.584 ± 9.711	201.318 ± 17.740	−25.249 ± 0.837	−129.919 ± 17.381
PI3K	Native ligand	−85.492 ± 18.675	−98.064 ± 57.119	176.346 ± 74.158	−12.801 ± 2.094	−20.011 ± 23.103
	Se02	−182.499 ± 19.445	−30.823 ± 24.734	150.574 ± 47.547	−20.019 ± 1.483	−82.767 ± 21.369
NF-ΚB	Native ligand	−139.618 ± 11.007	−34.531 ± 25.211	120.493 ± 26.547	−15.909 ± 0.639	−69.565 ± 13.830
	Se03	−238.397 ± 15.323	−55.390 ± 18.886	219.017 ± 26.684	−24.322 ± 1.095	−99.091 ± 17.208

**Table 8:** Elution system for the separation of the jengkol extract using HPLC.

T (min)	% ACN	% Water	Gradient Steepness (%/min)
0	5	95	1
5	5	95	1
30	95	5	1
35	95	5	1
38	5	95	1
40	5	95	1

**Table 9:** Structure of organic Se prediction in jengkol.

No.	IUPAC Name	Structure
1.	Compound Se01 Selenomethionine (SeMet)	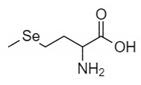
2.	Compound Se02 Glutamyl-glycinyl-N-2,3-DHP-selenocysteine (Gamma-Glu-MetSeCys)	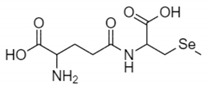
3.	Compound Se03 Se-S conjugate of cysteine-selenoglutathione	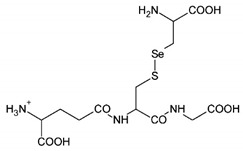

## Data Availability

Data is contained within the article.
